# Evolutionary crossroads of cell signaling: PP1 and PP2A substrate sites in intrinsically disordered regions

**DOI:** 10.1042/BST20200175

**Published:** 2021-06-08

**Authors:** Bernhard Hoermann, Maja Köhn

**Affiliations:** 1 Signalling Research Centres BIOSS and CIBSS, University of Freiburg, Schänzlestraße 18, 79104 Freiburg, Germany; 2 Faculty of Biology, University of Freiburg, Schänzlestraße 18, 79104 Freiburg, Germany

**Keywords:** 14-3-3 proteins, intrinsically disordered proteins, protein phosphatases, protein-serine-threonine kinases, substrate recognition

## Abstract

Phosphorylation of the hydroxyl group of the amino acids serine and threonine is among the most prevalent post-translational modifications in mammalian cells. Phospho-serine (pSer) and -threonine (pThr) represent a central cornerstone in the cell's toolbox for adaptation to signal input. The true power for the fast modulation of the regulatory pSer/pThr sites arises from the timely attachment, binding and removal of the phosphate. The phosphorylation of serine and threonine by kinases and the binding of pSer/pThr by phosphorylation-dependent scaffold proteins is largely determined by the sequence motif surrounding the phosphorylation site (p-site). The removal of the phosphate is regulated by pSer/pThr-specific phosphatases with the two most prominent ones being PP1 and PP2A. For this family, recent advances brought forward a more complex mechanism for p-site selection. The interaction of regulatory proteins with the substrate protein constitutes a first layer for substrate recognition, but also interactions of the catalytic subunit with the amino acids in close proximity to pSer/pThr contribute to p-site selection. Here, we review the current pieces of evidence for this multi-layered, complex mechanism and hypothesize that, depending on the degree of higher structure surrounding the substrate site, recognition is more strongly influenced by regulatory subunits away from the active site for structured substrate regions, whereas the motif context is of strong relevance with p-sites in disordered regions. The latter makes these amino acid sequences crossroads for signaling and motif strength between kinases, pSer/pThr-binding proteins and phosphatases.

## Introduction

To react and adapt to changes, cells have developed more than 400 different post-translational modifications (PTMs), which act as dynamic modulators upon attachment to amino acid (AA) side chains in order to modify protein stability, activity, structure, localization and binding [1]. Among these, phosphorylation on the AAs serine (Ser) and threonine (Thr) is to date among the most frequently identified PTMs [1–3] and controls crucial cellular signaling events in processes such as learning, muscle contractility, growth, adaptation to stress, nutrient processing and immunity [4]. In multiple central signaling pathways aberrant phosphorylation on Ser/Thr represents a hallmark in the origin and manifestation of diseases and is therefore in the center of medicinal chemistry and drug development [5–7]. To provide the elementary understanding of signaling processes for future medical applications, a deep understanding of pSer/pThr regulation is therefore of central importance.

Advances in phosphoproteomics have led to a drastic increase in phosphorylation site (p-site) annotations over the last 15 years: Whereas at the beginning of the millennium the majority of the known phosphoproteome still consisted of p-sites identified and characterized by directed low-throughput (LTP) efforts aiming to study the role of single p-sites [8], this picture changed concomitant with the enormous speed of technological progress in subsequent years. This development is well reflected in a recent study by Beltrao and collaborators, combining knowledge of more than 6000 phosphoproteome experiments executed across human cell types. This holistic analysis of 112 human phosphoproteome datasets led to >119 000 phosphosites on ∼12 000 proteins (90 443 sites, when only considering high-confidence class I sites). These numbers therefore represent the to date most complete view on the human phosphoproteome [3].

Among all p-sites, pSer is observed significantly more often than pThr and pTyr: ∼5% constitute pThr, only 0.4–4% constitute pTyr sites, with pSer being by far more abundant [2]. When not only differentiating between classes of PTMs but also by AAs to which PTMs are attached to, pSer is therefore by far the most frequently observed PTM among all. Notably, these observations might also be influenced by methodological aspects, since PTMs vary in stability and detectability. However, despite having these large and precious datasets in hand, in-detail functional and regulatory characterization is lacking for the majority of these sites. Consequently, the increase in known p-sites currently does not lead to a linear increase in understanding of associated human diseases due to the lack of understanding of p-site regulation.

In this regard, many conclusions on pSer/pThr-sites were derived without considering the structural context surrounding pSer/pThr and the competing interplay between kinase, phosphatase and pSer/pThr-binding proteins. We see orthogonal insights into the regulation of the pSer/pThr phosphoproteome by the Phosphoprotein Phosphatases 1 and 2A (PP1, PP2A) that regulate the majority of pSer/pThr dephosphorylation events [9], as a current bottleneck for its interpretation. With this review we aim to highlight the necessity for an independent, structure-focused view on the human pSer/pThr phosphoproteome for a full understanding of its regulation by phosphoprotein phosphatases (PPPs).

## Enzymes and interactors of the pSer/pThr phosphoproteome

Phosphorylation on pSer and pThr for modulation of protein function, structure, localization and activity is not regulatory by itself, but rather needs attachment and removal in a timely and specific manner in order to exhibit its full power in cellular signaling. The biggest advantage lies in the speed by which cells can change and adapt protein function upon signal input [10]. Whereas adapting to input by transcriptional or translational changes implies a rather long, multi-layer process, the modification of expressed proteins allows fast adaptation. Yet, an increase in pSer/pThr sites for adapting to more and more complex signaling events implies an equally complex toolbox of modifying enzymes in order to maintain the advances in pSer/pThr signaling cascades. For Ser/Thr-specific kinases, multiple gene duplications coincide with the growth of the functional phosphoproteome, leading to site-specific, motif-dependent Ser/Thr-phosphorylation. These motifs can range from acidic for Polo-like kinases (PLKs) [11,12] to basic, such as the R-R-x-S/T consensus in the PKA kinase motif or the often found consensus S/T-P-X-K for cyclin-dependent kinases (CDKs) [13].

pSer/pThr-binding protein domains, such as WW, forkhead-associated (FHA), and 14-3-3, have also been identified to add complexity to phosphosignaling by recruiting partner proteins and serving as signaling hubs in a phosphorylation-dependent manner. For 14-3-3 proteins, the Mode 1 (RSX(pS/T)XP) and Mode 2 (RX(F/Y)X(pS)XP) consensus motifs of 14-3-3 binding sites [14,15], mostly in disordered protein regions, have been identified [16–18]. Therefore, these proteins can be considered an additional layer for amplification of complex pSer/pThr signaling and the interplay with regulating kinases is well-established [15,17,19,20]. Several thousand 14-3-3 binding sites have been predicted in high-throughput approaches, but detailed characterization is lacking [21].

What has not entered the equation and the more holistic view of pSer/pThr signaling, are the counteracting phosphatases. This is mainly due to the fact that the picture is less clear for the most prominent family of pSer/pThr phosphatases, namely the PPPs. This subfamily of phosphatases consists of 7 phosphatases: PP1, PP2A, PP2B (Calcineurin), PP4, PP5, PP6 and PP7, with two of its best-studied members being Protein Phosphatase 1 and 2A (PP1, PP2A), on which this review focuses on. For most members of this set of phosphatases, evolution chose a different path than for kinases. PPPs mostly consist of a highly conserved catalytic core protein. This catalytic subunit (denoted for PP1 and PP2A here as PP1c and PP2Ac) does not show high degrees of flexibility, but rather provides rigid binding interfaces for the temporary docking of a multitude of substrate-specifying regulatory subunits [22]. These attach to the catalytic core protein in a combinatorial manner to form dimeric or even trimeric holoenzyme complexes in order to create specificity [9,23]. Similar to the catalytic subunits for kinases, the PPP regulatory subunits have been subjected to evolutionary diversification, leading to hundreds of different holoenzymes that specifically dephosphorylate certain pSer/pThr substrate sites [22].

Early findings of the 1980s and 1990s suggested that PP1c and PP2Ac exhibit an intrinsic specificity towards certain substrate motifs. These observations at the time were based either on sets of peptides synthesized in a targeted manner [24,25] or on functional read-outs of PP1/PP2A-regulated ion channels [26,27] and were already under dispute at the time [28]. Despite these pieces of evidence, the predominant opinion that was established over the following decades was that whereas kinases have intrinsic motif preference for AA surrounding pSer/pThr, PP1c and PP2Ac do not have substrate preferences around the active site. However, this picture for PP1and PP2A substrate specificity is incomplete, since recent advances in phosphatase research combined with the picture from proteomic approaches widen the process for p-site recognition to a contribution from the PP1c and PP2Ac subunit that favors certain motifs surrounding pSer/pThr, similar to the findings for the counteracting kinases: In the holoenzyme scenario the motif context surrounding pSer/pThr played a role for substrate selection [29–32], and also the preference of the complex of the PP2A catalytic subunit with its B55 regulatory subunit for pThr over pSer overlapped with early observations [32–35]. Our laboratory has recently provided evidence that the preferences for basic residues *N*-terminal of the p-site for PP1 and for pThr over pSer is caused by the catalytic subunit itself [36]. Consequently, not only the regulatory subunits create specificity, but also the surface in close vicinity of the catalytic cleft interacts with AAs *N*- and *C*-terminal of the p-site, thereby creating specificity on the motif-level, and also the catalytic cleft creates specificity for pThr over pSer in the cases of PP1c and PP2Ac [31,36–38]. Indeed, also for PP2B recent evidence shows a role of the active site in substrate recognition [39].

## Intrinsically disordered regions as phosphorylation signaling hubs

In contrast to p-sites in ordered regions and Pfam domains, p-sites located within intrinsically disordered regions (IDRs) are located in sequence stretches and loop regions characterized by high flexibility that do not engage in stable secondary and tertiary conformations in their ground state. As a consequence, these IDRs are lacking structural features and binding interfaces that could potentially contribute to p-site selection for pSer/pThr-specific scaffold proteins, kinases, and phosphatases. Therefore, the p-site recognition in IDRs does not follow the classical thinking, in which secondary and tertiary protein structure determines the function of the protein region in a key/lock-like manner, also termed the function-follows-structure paradigm [40].

Given the two-layered system for p-site recognition by PP1 and PP2A holoenzymes introduced above, we hypothesize that these layers might contribute to p-site selection with varying degrees between structured and disordered regions. However, in the current literature there is a clear bias towards studying the regulatory impact of phosphorylation sites in ordered regions [41] due to two main factors: Firstly, residues in IDRs, due to their intrinsic flexibility, are often difficult to assign in structural biology methods, especially in crystallography, making it difficult to add structural insights to studies of these sites. Also, many cases are known for which an often unknown complex partner is needed in order to stabilize IDRs for structural studies [41]. Secondly, a large fraction of bioinformatic methods uses evolutionary sequence conservation in order to identify sites of regulatory importance. Yet, it has been clearly demonstrated in the case of cyclin-dependent kinase 1 (Cdk1) that despite their regulatory importance, p-sites in IDRs are more rapidly evolving and that the exact location of the p-site within IDRs is less relevant, making them more difficult to identify and study [42]. Due to the lower sequence conservation in IDRs, p-sites in these protein parts are less likely to be chosen for detailed follow-ups. Yet, studies from Iakoucheva et al. show the potential of studying p-sites in IDRs [43] and very recent work of Cho et al., in which they applied statistical thermodynamical approaches to understand the disordered phosphoproteome [44], clearly demonstrates the potential of analyzing kinase substrate sites in IDRs beyond considering solely kinase motif sequences.

**Figure 1.**
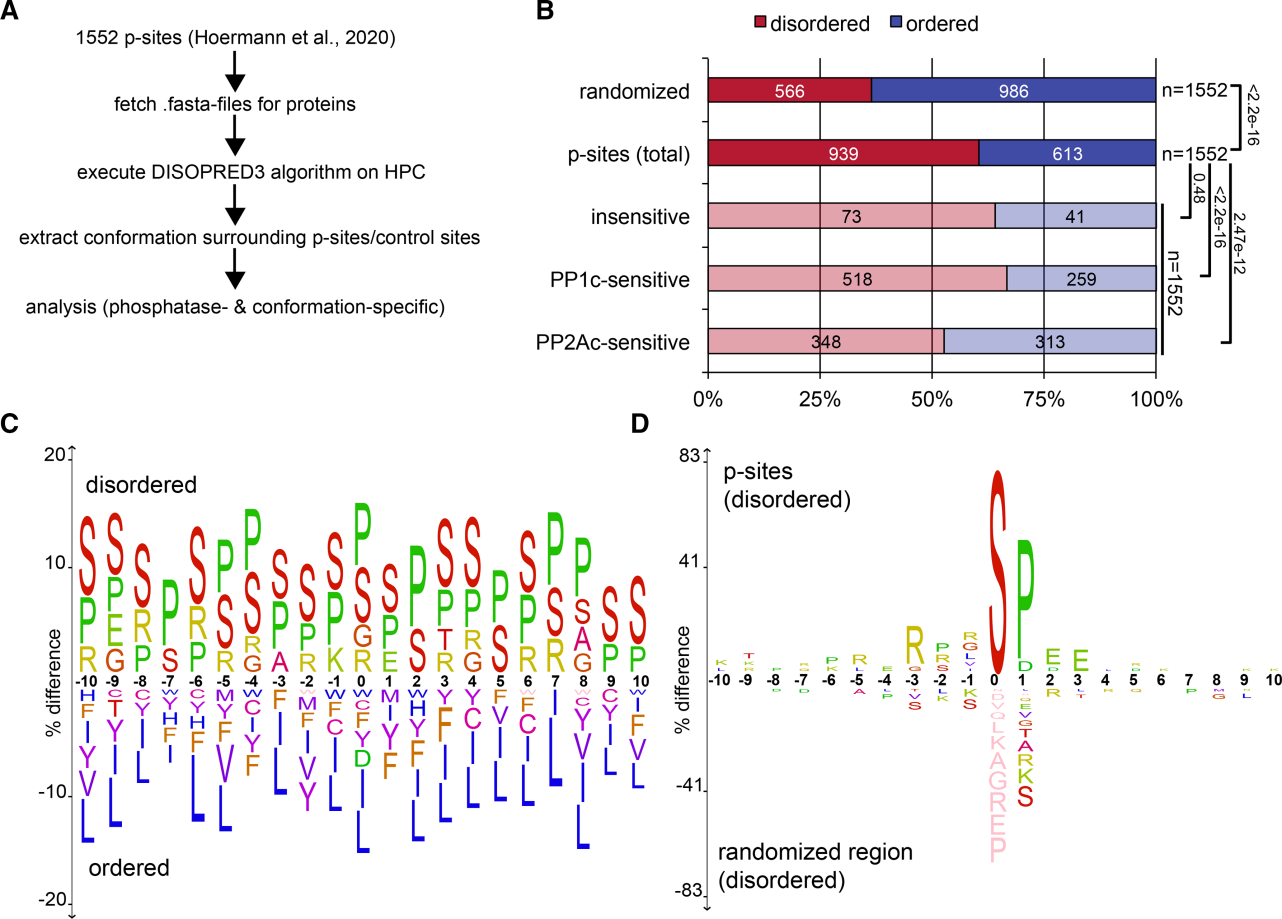
Analysis of a set of 1552 p-sites identified in Hoermann et al. [36] for structural properties. (**A**) Full length .fasta sequences of underlying phosphosites were retrieved from uniprot.org using the UniprotKB (accessed 11/12 2020) of the majority protein. IDRs were identified using the DISOPRED3 algorithm [48] (default settings, i.e. FDR 5%) on the bwUnicluster 2.0 high performance computing (HPC) resource of the state Baden-Wuerttemberg using batch scripts. The output .diso files were then further analyzed locally using python3 and R. (**B**) Information on p-site conformation was extracted from .diso DISOPRED3 output files, including +- 15 amino acids surrounding the p-site. Sites within 15 amino acids of the *N*- or *C*-terminus of the respective protein were included, inserting blank values for empty positions. A control dataset was generated by randomly selecting a 31 amino acid stretch on the same protein. A stringent definition for disordered regions was chosen: Only if all 30 amino acid residues surrounding the p-site were predicted to be disordered, a p-site region was considered disordered, otherwise these p-sites were assigned to the ordered category. This approach follows previous definitions for the identification of long IDRs [45,46]. The definition of PP1c or PP2Ac-sensitive sites follows the definition of Supplementary Data 2 in Hoermann et al. [36]. Statistics represent the p-value of a Fisher's exact test carried out in R. (**C** and **D**) The sequence motif within the categories depicted in Figure 2B were then further analyzed using iceLogo [78]. The sequence stretch submitted to motif analysis was limited to 10 AA *N*- and *C*-terminal of the p-site for better visualization and readability of motifs. The motifs were generated for a p-value of 0.05 and the amino acid labeling corresponds to the hydrophobicity at pH 7.0 with: blue = hydrophobic; red = neutral; green = acidic, charged; and yellow = basic, charged. (**C**) The motif comparison demonstrates the enrichment of amino acids S, P, R and K in the general amino acid distribution in IDRs. (**D**) Around p-sites in IDRs, R in position −3 and P in +1 are even further enriched.

To take an exemplary look at p-sites in long (≥31 AAs) IDRs [45,46] we analyzed phosphoproteomic datasets from our group [36] consisting of 1552 pSer/pThr sites. To this end, the full sequences of all corresponding proteins were retrieved from uniprot.org [47] and analyzed by DISOPRED3 [48], a reliable algorithm for identification of IDRs [49], using high-performance computing (HPC, Figure 1A). In our own dataset of 1552 p-sites, 939 (61%) of these were located in long IDRs [45,46], providing evidence that p-sites in IDRs are a common scenario in cellular signaling (Figure 1B). Further support that a distinct look on the IDR-located part of the phosphoproteome might lead to interesting insights comes from the observation that the phosphorylation state of an IDR seems to correlate with IDR length [50].

Although IDRs themselves do not exclude the possibility that secondary and tertiary structure more distant from the p-site contribute to p-site selection, the intrinsic features of IDRs give the primary amino acid sequence in direct vicinity of pSer/pThr central importance and increased relevance for determining selectivity. The importance of p-sites in IDRs has been demonstrated in very recent proteomic approaches using thermal proteome profiling and pulse-chase-based phosphoproteomic experiments: In both cases the effect of phosphorylation on protein stability was largest with p-sites located in IDRs compared with structured regions, providing evidence that a large fraction of p-sites in IDRs serves a regulatory function [51,52]. Also for phosphatases, an enrichment of substrate sites in IDRs has been postulated, since most known substrate sites for phosphoprotein phosphatases PP1, PP2A, PP2B and PP2Cα are located in unstructured regions [53]. This trend is supported by the presented data for PP1, since despite being already enriched in IDRs, p-sites sensitive to PP1c are even more enriched in IDRs (Figure 1B).

While amino acid composition alone is not sufficient to identify and classify IDRs [54], work by Iakoucheva et al. and Basile et al. demonstrated that IDRs are enriched in the AAs Ser, Pro, Lys [55] and potentially Arg [43]. These findings are well supported by our own analysis, showing that in our dataset Ser, Pro, Arg and to some degree Lys are enriched by up to 15% in IDRs, whereas hydrophobic AAs are found more frequently in structured protein regions (Figure 1C). When looking at p-sites located within IDRs in our exemplary dataset, an even higher enrichment for Pro in +1, as well as for Arg in -3 is observed compared with the general AA distribution in IDRs. Therefore, these features can be considered p-site-specific (Figure 1D). Importantly, these enrichments also represent characteristic features of kinase- and 14-3-3-motifs introduced previously.

## The role of IDRs in the context of PP1/PP2A holoenzymes and substrates

There is solid evidence for the importance of IDRs in interacting with the rigid catalytic core of PP1 and PP2A: For PP1, it has been demonstrated that more than 70% of its regulatory proteins constitute intrinsically disordered proteins (IDPs). For example, the complex of PP1 with its myosin-targeting subunit MYPT1 (PP1:MYPT1) [56], spinophilin [57], inhibitor 2 (I-2) [58] and Phosphatase 1 NUclear Targeting Subunit (PNUTS) [59–61], rely on disordered regions, termed short linear motifs (SLiMs) in order to interact with the catalytic subunit [62]. These (partially) disordered proteins adapt to the rigid PP1 catalytic core protein (PP1c), thereby following the paradigm that in most protein–protein interactions one binding partner provides a rigid template to which a second flexible partner protein adapts [62]. Interestingly, this rule also holds true in a reversed scenario for the interaction of PP1 with apoptosis stimulating protein of p53 (ASPP) for one of the only two disordered regions of PP1c, namely its C-terminal regulatory tail. The tail binds to the rigid SH3 domain of ASPP leading to its stabilization [63]. Similarly, for the complex of PP2A with its regulatory B56 subunit (PP2A:B56) an intrinsic preference of B56 to disordered regions in substrate proteins was found [29], for example within the B56 binding region of anaphase-promoting complex subunit 1(APC1) [64]. PP2A:B56 interacts with substrates by a SLiM consisting of an LxxIxE motif [29,65]. Also, the B55 subunit selects PP2A substrates by interaction with basic AAs in SLiMs around the p-site [66]. Therefore, despite the fact that in contrast to PP1 most regulatory B subunits of PP2A are rather structured, disordered regions are of great importance for substrate recruitment. Furthermore, very recent efforts allow to the widen angle and impact of this rational to other PPPs such as PP2B [67] and PP4 [68], which also use SLiM binding for substrate recognition making this a broad feature of the family.

**Figure 2.**
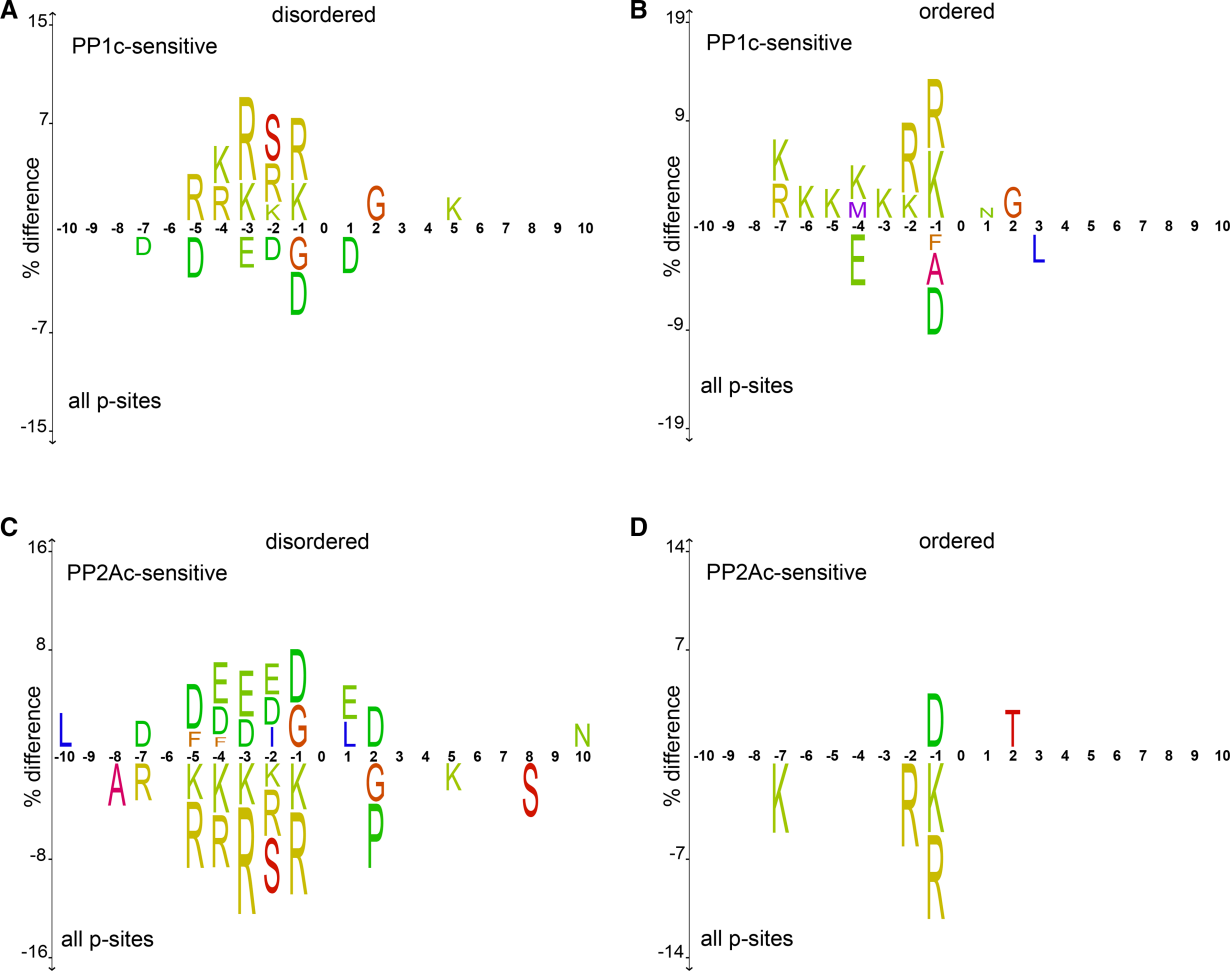
PP1c- and PP2Ac-sensitive sites display diverging motifs depending on their structural properties. (**A**) PP1c-sensitive sites are enriched for the often-found RxxpSP motif in IDRs. (**B**) The trend observed for PP1c in Figure 2A is not the case for ordered regions. In this scenario, the amino acid in −1 seems to be of higher importance compared with −3. (**C**) PP2Ac has a strong preference for acidic amino acids in disordered protein regions but disfavors basic residues R and K. (**D**) The trend observed for IDRs and PP2Ac in Figure 2C is not found in ordered regions. As for PP1c, the biggest effects are observed for the amino acid residues closest to the p-site.

It is therefore an intriguing idea to extend this paradigm to the regions on substrate proteins dephosphorylated by PP1c and PP2Ac: Since many of the PP1c and PP2Ac sensitive p-sites are located in IDRs lacking structural features for surface recognition, the primary amino acid context surrounding pSer/pThr gains special importance for interacting with the rigid grooves surrounding the catalytic cleft of these phosphatases. This places a layer of PPP specificity at the same level as motifs of kinase and pSer/pThr-binding proteins and makes these amino acid sequences crossroads for signaling and motif strength between enzyme classes. In fact, several cases are known for such a scenario: PP1/Repo-Man regulates the highly disordered *N*-terminus of Histone 3 (H3) [69,70] by dephosphorylating T3 [60] and S10 [71,72] to regulate mitosis, which in turn regulates the association of 14-3-3 proteins [73]. Similarly, the intrinsically disordered region around S455 of IRSp53 is a well-established substrate of the PP1/Phactr complex [74], which is also bound by 14-3-3 proteins [75]. Also, during entry into M-phase, CDC25C gets dephosphorylated by PP1 in its unstructured region around pS216 [76], again a site normally occupied by 14-3-3 proteins [77]. Indeed, we find in our own data that p-sites sensitive to PP1c and PP2Ac differ, depending on their structural characteristics: For PP1c, we find that the enrichment of Arg in −3 is only important in disordered regions, whereas position −1 plays a more important role in ordered domains (Figure 2A,B). For PP2Ac, we see that in general the impact of positive or negative charges is of higher relevance in the flexible loops of IDRs compared with structured regions (Figure 2C,D). In both cases this suggests that in flexible IDRs the impact of AA around the p-site is of higher relevance compared to p-sites in structured regions.

**Figure 3.**
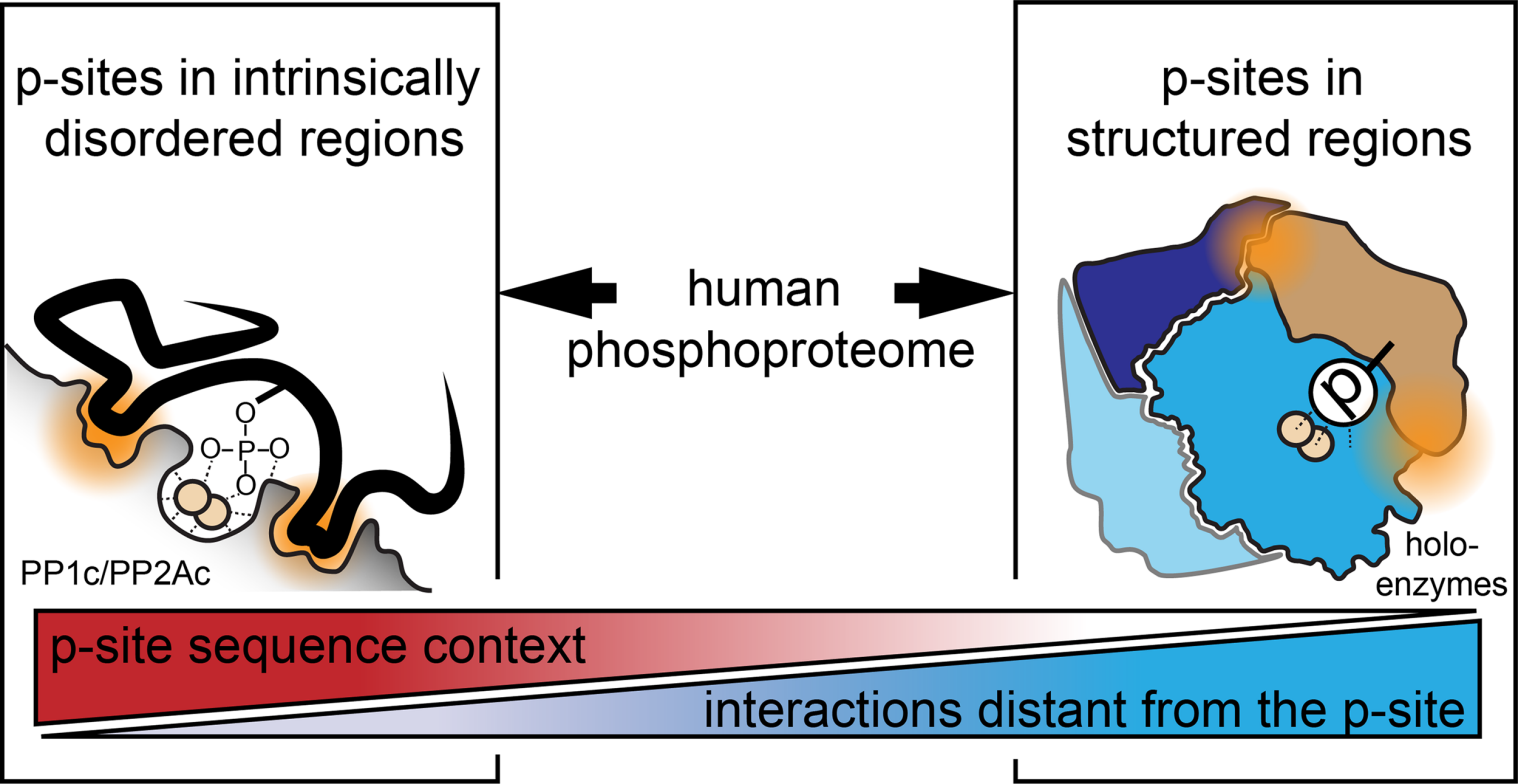
Scheme displaying the potential impact of p-site structure on the dephosphorylation mechanism. According to this model, for PP1 and PP2A-sensitive p-sites located in IDRs, the amino acid sequence surrounding the p-site is important. The highly flexible region is able to align with the surface structures including grooves surrounding the catalytic cleft of PP1 and PP2A, giving the charges surrounding pSer/pThr higher importance compared with p-sites in ordered regions and adding a layer of p-site motif-dependent recognition and competition between phosphatases, kinases, and pSer/Thr binding proteins. In the latter case, active site recognition is less important and protein–protein interactions further away, for example between regulatory and catalytic subunit and the substrate protein, determine the substrate recognition.

Altogether, the following model of PP1/PP2A substrate specification has evolved: As a first layer for substrate targeting, PP1 and PP2A associate with regulatory subunits. This step can also lead to binding of a regulatory subunit close the catalytic subunits’ surface surrounding the catalytic cleft, which in turn alters the motif preference of the holoenyzme. The second layer of p-site selectivity is determined by the catalytic subunit and its surface properties as such and is actively contributing to the overall specificity of the holoenzyme [32]. These layers likely differ in strength and functionality depending on the regulatory subunit as well as the structural context of the p-site. Also, as recently demonstrated for kinase Ser-Pro (SP) motifs, mechanisms underlying the recognition of these p-sites are expected to involve thermodynamic factors, such as differences in free energy between phosphorylated/phosphorylated states [44]. Based on the above, we hypothesize that the second layer on the catalytic subunit level might be especially suited for binding IDRs and recognizing primary amino acid motifs around pSer/pThr, and might represent the main contribution for competing binding affinities between kinases, phosphatases and pSer/pThr-binding domains (Figure 3). This view is in good consent with models proposed by Rogers et al. that had not yet considered differences in amino acid distribution between IDRs and structured regions [38]. Based on recent findings regarding the substrate specificity of the catalytic subunits of PP1 and PP2A, we propose that the recognition of the AAs around the active site by the catalytic subunit could be a more important feature for PP1 than for PP2A.

## Conclusion

The success in interpreting the exact role of more than 100 000 human p-sites in health and disease is largely dependent on deciphering a complex system between kinases, p-motif binding proteins and the multilayer specificity of phosphatases in the light of motif context and structural information. Given the recent insights presented in this review, we anticipate that disentangling the phosphoproteome in terms of sequence context and structural features will strongly help to elucidate the regulatory relevance of p-sites and the mechanism of p-site recognition by PP1 and PP2A in the upcoming years, and will support completing the understanding of the interplay between kinases, phosphatases, and interacting proteins with phosphoprotein p-sites.

## Perspectives

- Whilst data for the human phosphoproteome currently comprises more than 100 000 identified phosphorylation sites, only a small fraction has been studied in terms of regulation by kinases, phosphorylation-dependent binding proteins, and phosphatases.
- The importance for the sequence context of substrate sites for Ser/Thr kinases and pSer/pThr-binding proteins is quite well established, however, a more complex model for the pSer/pThr phosphatases PP1 and PP2A is currently evolving.
- For these phosphatases, further studies are needed to differentiate between substrate recognition by regulatory subunits distant from the pSer/pThr substrate site and motif recognition in direct vicinity of the pSer/pThr by the catalytic subunit. We hypothesize that these layers affect substrate recognition differently in structured and disordered protein regions.

## Abbreviations

AA: amino acids
ASPP: apoptosis stimulating protein of p53
HPC: high-performance computing
IDRs: intrinsically disordered regions
PPPs: phosphoprotein phosphatases
PTMs: post-translational modifications
SLiMs: short linear motifs
